# Sedentary Behaviour and Fall-related Injuries in Aging Adults: Results from the Canadian Longitudinal Study on Aging (CLSA)

**DOI:** 10.14283/jarlife.2024.14

**Published:** 2024-07-17

**Authors:** M. Gallibois, C. Hennah, M. Sénéchal, M.F. Fuentes Diaz, B. Leadbetter, D.R. Bouchard

**Affiliations:** 1. Cardiometabolic Exercise and Lifestyle Laboratory, Faculty of Kinesiology, University of New Brunswick, Canada; 2. Faculty of Kinesiology, University of New Brunswick, Canada; 3. School of Psychology, Queen’s University Belfast, Belfast, United Kingdom

**Keywords:** Sedentary behaviour, Fall-related injuries, CLSA

## Abstract

**Background:**

Falls, and more specifically, fall-related injuries, are costly to the healthcare system and can harm one’s autonomy.

**Objectives:**

To study the impact of sedentary behaviour associated with fall-related injuries and how a change in sedentary behaviour may impact the risk of a fall-related injury.

**Design:**

From baseline to the first follow-up, cross-sectional and longitudinal data analysis from the Canadian Longitudinal Study of Aging (CLSA) cohort.

**Participants:**

CLSA data from 43,558 Canadians aged 45-85 were included in this study.

**Measurements:**

At baseline and follow-up, sedentary behaviour time was categorized as low (<1,080 minutes/week), moderate (1,080-1,440), or high (>1,440). Sedentary behaviour was estimated via the Physical Activity Scale for the Elderly (PASE). At follow-up, participants were dichotomized as either increased or decreased/no change in sedentary behaviour according to their categorical change between time points.

**Results:**

Sedentary behaviour was associated with fall-related injuries independently of age, sex, number of chronic conditions, and total physical activity levels OR (95%CI) 1.10 (1.05-1.15). In contrast, a change in sedentary behaviour was not associated with the risk of fall-related injury 1.00 (0.92-1.01).

**Conclusion:**

A higher level of sedentary behaviour is associated with injurious falls for people between 40 and 80 years old. However, a short-term change in sedentary behaviour does not influence the risk of injury-related falls. Despite the results, a more precise measure of sedentary behaviour is needed for epidemiology studies to capture changes over time better.

## Introduction

**F**alls are the second leading cause of injury deaths worldwide (1), contributing to 80% of all injury-related hospitalizations (2). Fall-related injuries are estimated to cost ~$10,000 per person (3), largely due to lengthy hospital stays. They are complex and lead to long-term health consequences such as chronic pain, social isolation, and disability (4). An estimated 30% of community-dwelling older adults (65+) fall annually, from which one in three are injured (5). Most fall research has focused on older adults, but middle-aged adults also experience a high incidence rate of falls and fall-related injuries (6). Including middle-aged adults in fall prevention, research offers insight into early risk factor identification and reduction. The Baltimore Longitudinal Study of Aging found that 21% of middle-aged adults reported falling in the past two years (7). Middle-aged adults may experience high fall rates simply because they are more active than older adults (8) and as biological and behavioural risk factors increase, including sedentary behaviour (7). Sedentary behaviour includes any waking behaviour characterized by low energy expenditure while sitting, reclining, or lying (9).

A common misconception is that sedentary behaviour and physical inactivity can be used interchangeably. However, physical inactivity is used for a person who does not meet the recommended physical activity guidelines of 150 minutes of moderate to vigorous physical activity per week (9). The average adult in North America spends 9-10 hours of their day engaging sedentary behaviour (10) and many do not reach the physical activity guidelines. Less known is that even if one is active, the risk factor related to sitting too much is not cancelled (11). As a result, there is a growing interest in the health benefits of simply sitting less (i.e., reducing sedentary behaviour).

In fact, independent of physical activity, sedentary behaviour is associated with many negative health outcomes, such as chronic conditions and death (12–14). Sedentary behaviour exacerbates the risk of falls with aging because of its association with reduced bone mass (15), muscle mass and strength (16), which may increase the fall risk as one ages. However, the relationship between sedentary behaviour and fall-related injuries is inconsistent. Multiple studies have found that sedentary time is associated with increased falls among older adults (17, 18). It is suggested that sedentary behaviour can reduce physical function components which may lead to an increase in falls. On the contrary, increased sedentary behaviour may be protective as it can reduce the exposure and opportunity for falls to occur. A study by Bea et al. (2013) found that people displaying a highly sedentary lifestyle were less likely to experience a fall (19), Which could be due to the tool used to capture the exposure. Sedentary behaviour can be measured through wearable devices such as accelerometers and inclinometers—the latter is the gold standard. However, these tools are costly, making large-scale studies difficult. Sedentary Behaviour can also be measured subjectively through self-reported questionnaires. While questionnaires tend to have lower validity, they are cost-effective tools more commonly used in large cohort studies (20).

As such, more research is needed to identify the relationship between sedentary behaviour and falls. Provided that sedentary behaviour is potentially a modifiable risk factor for fall related injuries, there is a need to explore this relationship further. This study aimed to investigate the association between sedentary behaviour and fall-related injuries and explore whether changes over 18 months are associated with fall-related injuries.

## Methods

### Study Design and Participants

The participants were drawn from the Canadian Longitudinal Study on Aging (CLSA) (22). This Canada-wide study includes a representative sample of 51,338 community-dwelling adults aged between 45 and 85 when recruited. The objective of the CLSA is to perform tests on the cohort every three years for 20 years. The CLSA comprises a comprehensive (n=~30,000) and tracking cohort (n=~20,000). Data collection for participants in the tracking cohort is conducted through telephone interviews only, while participants in the comprehensive cohort undergo additional physical assessments at data collection sites (23). Excluded from CLSA are residents in the three territories and some remote regions, persons living on federal First Nations reserves and other First Nations settlements in the provinces, full-time members of the Canadian Armed Forces, and individuals living in institutions. Data from baseline and follow-up collection points were included. Of the 51,338 participants in the CLSA, 7,780 were excluded due to missing data related to falls or sedentary behaviour levels at baseline and/or follow-up. Therefore, 43,558 participants were included in the study for the cross-sectional and longitudinal analyses.

All participants provided written consent, and this study was approved by the Research Ethical Board Review Committee (REB approval # 2020/050).

### Measurements

#### Main outcome: Fall-related Injuries

Fall-related injuries were self-reported at baseline and follow-up via structured interview questionnaires. At baseline, participants were asked to respond (yes or no) to if they had a fall-related injury in the past 12 months. At follow-up, a 2-step procedure was used to derive fall-related injuries. First, participants were asked, “In the last 12 months, have you had any injuries that limited some of your normal activities? For example, a broken bone, a bad cut or burn, a sprain, or a poisoning”. Second, if the participant answered “Yes” to the question, they were then prompted, “Was this injury (were any of these injuries) caused by a fall?” At follow-up, three years after, incidences of fall-related injury in the past 12 months were dichotomized into a yes/no response as the outcome variable.

#### Exposure: Sedentary Behaviour

Sedentary behaviour is operationalized as self-reported sitting time. In the CLSA, the modified Physical Activity Scale for the Elderly (PASE) questionnaire was used to measure sedentary behaviour after 18 months from baseline and at the 3-year visit. Participants were asked to report their frequency and duration of sitting time over the past seven days. Because of the nature of the questionnaire, it is impossible to treat the exposure as a continuous variable. In addition, the distribution was skewed. To capture the weekly frequency of sedentary behaviour, participants were asked, “Over the past seven days, how often did you participate in sitting activities such as reading, watching TV, computer activities, or doing handcrafts?”. Possible responses were: Never (0); seldom (1 to 2 days); sometimes (3 to 4 days); and often (5 to 7 days)”. To measure daily duration, they were asked: “On average, how many minutes per day did you engage in these sitting activities?”. Possible responses to this question were: less than 30 minutes; more than 30 minutes but less than 1 hour; more than 1 hour but less than 2 hours; more than 2 hours but less than 4 hours and 4 hours or more. The mid-point of each frequency and duration category (except for the “four hours or more” duration category, where four was used) was used to estimate weekly sedentary time by their product. Due to the high prevalence of sedentary behaviour and the nature of the questions, the distribution was skewed, with 38.0 % of the sample maximizing the score at baseline and 47.7% at follow-up. As a result, according to total sedentary time, participants were categorized into three weekly levels of sedentary behaviour:

- Low: Less than 1080 minutes with different combinations of frequencies/times
- Medium: 1080 minutes (3-4 days per week for 4 hours or more)
- High: 1440 minutes (5-7 days per week for 4 hours or more).

Change in sedentary behaviour was categorized into two different categories:

- Increased sedentary behaviour: Greater category at follow-up compared with baseline (low to medium; medium to high).
- Decreased or no change: Lower category at follow-up compared with baseline or no change in sedentary behaviour.

#### Covariates

All variables were collected for both cohorts except medication and measured with the tracking cohort only. Age, sex, ethnicity, educational level, and marital status were self-reported via a questionnaire at baseline. The number of chronic conditions was quantified by asking participants if they had chronic conditions diagnosed by a healthcare provider. The chronic conditions included in our analysis were osteoarthritis of the knee, osteoarthritis of the hip, rheumatoid arthritis, osteoporosis, chronic obstructive pulmonary diseases, cardiovascular disease, Parkinson’s, multiple sclerosis, dementia (including memory problems), kidney disease, diabetes, incontinence (bowel and urinary), and mood disorder. These conditions are associated with falls (24–27).

Participants were asked to recall how often and how many prescription medications they took in the past month. The product of frequency and number of medications was computed into the average number of medications per week. Methods for collecting height and weight to calculate body mass index (BMI) were different between cohorts. Participants in the tracking cohort were asked to self-report their height and weight, which were objectively measured by trained professionals in the comprehensive cohort.

The PASE was used to estimate moderate to vigorous physical activity. Aerobic physical activity was defined as self-reported moderate and strenuous activities in the past seven days. Participants were asked to estimate the frequency and duration of engagement in each moderate and strenuous training activity. Possible responses for frequencies were Never (0 days), Seldom (1 to 2 days), Sometimes (3 to 4 days), and Often (5 to 7 days). Possible answers for the duration were: Less than 30 minutes; More than 30 minutes but less than 1 hour; More than 1 hour but less than 2 hours; More than 2 hours but less than 4 hours; 4 hours or more. The midpoint of each frequency and duration category (except for the 4 hours or more duration category) was multiplied to estimate weekly totals. The weekly totals for moderate and strenuous time were summed together in minutes for aerobic physical activity. To be used in the study’s models, aerobic physical activity was categorized according to whether participants reported spending zero minutes or greater than zero minutes engaging in aerobic activity.

The last steps were repeated for resistance training. The frequency and duration of resistance training were computed into weekly total time and then dichotomized as doing some resistance training in the past week (1) or none (0).

### Statistical Analysis

Differences between sedentary behaviour groups (low, medium, high) were investigated using analysis of variance (ANOVA). Logistic regression models were developed to investigate the odds of a fall-related injury based on sedentary behaviour categorization at baseline and changes in sedentary behaviour categorization between baseline and follow-up while adjusting for covariates using the ‘low sedentary group’ as the control group. Three sets of analyses were performed. First, the covariates used in the models were: Model 1 = Unadjusted; Model 2: Adjusted for age; Model 3: adjusted for age and sex; Model 4: adjusted for age, sex, chronic conditions, moderate to vigorous physical activity, and resistance training. The 4-step model was repeated while adjusting for the same potential confounders to investigate the association of fall-related injuries with increased sedentary behaviour. In addition, participants who fell at baseline were removed from the model. Subgroup analyses were performed by separating middle-aged and older aged adults. All data were analyzed using IBM SPSS Statistics (Version 27) and SAS.

## Results

A total of 43,558 participants from the CLSA were included in our analysis (Table 1). Sixty percent of participants were middle aged (45-65 years), 48.8% were male, and more than 95% were White Caucasian. The average BMI was classified as overweight (BMI between 25 and 29.9), with 1/3 of participants reporting at least one chronic condition. The participants classified as having high sedentary behaviour (n= 16,555, 38.0%) were significantly older, heavier, reported more chronic conditions, did less exercise, and reported more fall-related injuries than those classified as low sedentary behaviour. At the baseline, 4813 (11%) participants reported a fall-related injury in the past 12 months, while only 2953 (6.8%) participants reported a fall-related injury at follow-up.

**Table 1.** Descriptive Characteristics of Participants (n=43,558)

	Sedentary Behaviour Categorization		
	Low (<1079 min/week) n=9691 (22.25%)	Medium (1080-1439 min/ week) n=17312 (39.74%)	High (≥1440 min/week) n=16555 (38.01%)
Sex (Female)	4879 (50.3)	8757 (50.6)	8560 (52.3)
Age	59.91 + 10.09	62.72 + 10.06	64.08 + 10.04
Body Mass Index (kg/m2)	27.35 + 2.20	27.81 + 3.81	28.90 + 3.16
Ethnicity (white)	9283 (95.8)	16737 (96.7)	15968 (96.5)
Chronic Conditions (#)			
Zero	3883 (40.1)	5780 (33.4)	4369 (26.4)
One	2934 (30.3)	5247 (30.3)	4732 (28.6)
Two	1516 (15.6)	3031 (17.5)	3241 (19.6)
Three or more	1358 (14.0)	3254 (18.8)	4213 (25.4)
†Medication (#/week)	9.69 + 8.77	11.38 + 8.76	13.03 + 8.66
Marital Status (married)	7108 (73.3)	12531 (72.4)	10889 (65.8)
Education (University)	2400 (43.4)	7392 (42.7)	7052 (42.6)
No aerobic activities	5038 (52.0)	10028 (57.9)	10971 (66.3)
No resistance training	6563 (67.7)	12175 (70.3)	12145 (73.4)
Fall-related injury (yes)	976 (10.1)	1748 (10.1)	2086 (12.6)

Data presented as mean + SD or N (%); BMI = Body mass index; † = Only data from Tracking cohort (n=21,000).

**Figure 1.**
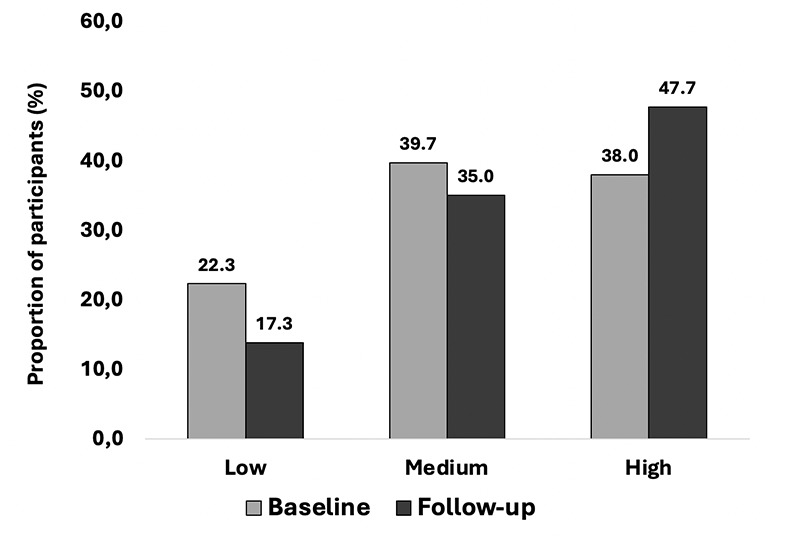
Distribution of participants within sedentary behaviour groups (Low-Medium-High) n=43,558

At baseline, 22.3% of participants were classified as having a ‘low level’ of sedentary behaviour, with that number decreasing to 17.3% at follow-up (Figure 1). Inversely, 9.7% of participants classified as ‘high level’ of sedentary behaviour went from 38.0% to 47.7%.

**Figure 2.**
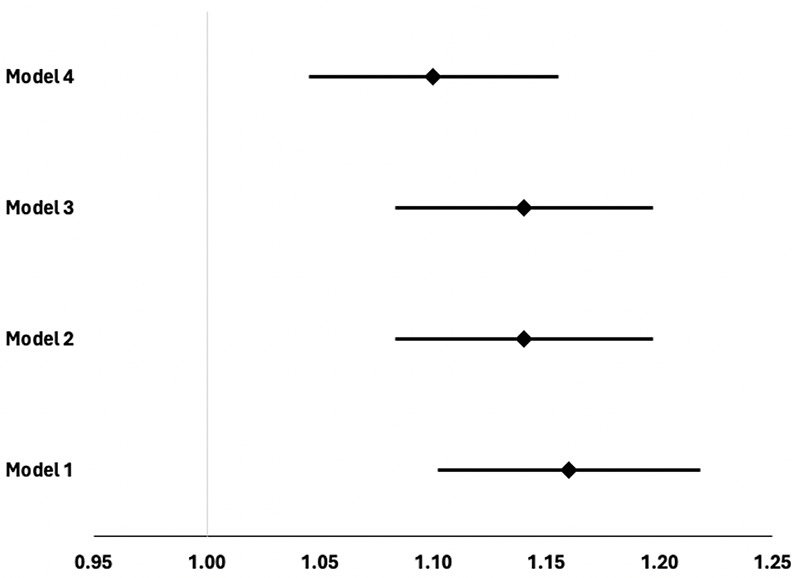
Association between injurious falls and sedentary behaviour at follow-up

Logistic regression investigated the association between injurious falls and sedentary behaviour at baseline. When the model was fully adjusted (age, sex, chronic conditions, moderate-to-vigorous physical activity, and resistance training), the odds of injurious falls increased by 1.10 (1.05 – 1.15) for each categorical increase in sedentary behaviour (Figure 2).

Data are presented as RR (95% CI). Model 1 = Unadjusted; Model 2: Adjusted for age; Model 3: Adjusted for age and sex; Model 4: Adjusted for age, sex, chronic conditions, moderate to vigorous physical activity, and resistance training.

Findings from the logistic regression model investigating the association between injurious falls and an increase in sedentary behaviour between baseline and follow-up showed that the odds of injurious falls were not significant,1.00 (0.92-1.01). When the model was fully what? Adjusted? (age, sex, chronic conditions, moderate-to-vigorous physical activity, and resistance training) (Table 2). Additionally, when participants who reported injurious falls at baseline were removed, the odds of fall-related injuries were 1.05 (0.93-1.12) when adjusting for the same confounders.

**Table 2.** Association between change in sedentary behaviour and fall-related injuries

	Relative Risk (95% CI)
Model 1- Unadjusted	0.99 (0.90- 1.10)
Model 2 – Adjusted for age	1.01 (0.91-1.11)
Model 3 – Further adjusted for sex	1.01 (0.92-1.12)
Model 4- Further adjusted for chronic conditions, moderate to vigorous physical activity, and resistance training.	1.00 (0.92-1.01)

Model does not included people who reported a fall with injury at baseline

A subgroup analysis of middle-aged and older adults yielded similar results. Cross-sectionally, the logistic regression model showed a significant association between falls and sedentary behaviour for middle-aged 1.11 (1.05-1.17) and older adults 1.09 (1.02-1.17). However, a change in sedentary behaviour was not significantly associated with falls in any of he groups 1.02 (0.91-1.15) for middle-aged and 0.95 (0.84-1.09) for older adults.

## Discussion

This study investigated the association between sedentary behaviour and fall-related injuries and whether sedentary behaviour changes impacted the risk of injurious falls. Our results revealed that sedentary behaviour was associated with fall-related injuries in the CLSA cohort of middle-aged and older adults independent of age, number of medical conditions, and physical activity. However, changes in sedentary behaviour between baseline and follow-up testing did not lead to a change in relative injury fall risk.

Considering the primary aim of this study, these results were consistent with those found in the systematic review by Semanik et al. (28), where sedentary behaviour was associated with increased falls. This study shows that this relationship exists independently of physical activity in both men and women. This could have significant implications for maintaining the health and well-being of middle-aged and older adults, as this study highlights the potential harms of sustained sedentary behaviour. High sedentary behaviour could also identify those most at risk of fall-related injuries in middle-aged and older adults.

Interestingly, our results are inconsistent with a similar study performed by Lustosa et al. (14), who found no association between sedentary time and fall-related injuries despite an association between sedentary time and self-reported falls. However, their study used a median split method, which only allowed for sedentary time to be classified as one of two (low or high) sedentary time categories. In contrast, the present study used a three-category model where participants were classified as having either low, moderate, or high levels of sedentary time. These findings reinforce the dose-response relationship between sedentary behaviour and health outcomes and highlight the necessity for a continuous measure of sedentary time rather than a categorical approach.

While the results of this paper confirmed the main hypothesis, exploring our secondary aim yielded surprising results, as categorical changes in sedentary behaviour (between baseline and follow-up) were not associated with a change in relative fall risk. This is incongruent with our primary finding that sedentary behaviour is associated with falls. In addition, our results challenge the work of Bea et al. (2017), who found that fall risk did increase with a reduction in sedentary behaviour in a female sample over six years (19). A longer period of sustained reduction in sedentary behaviour may be required to see the benefits. From this perspective, it is reasonable to conclude that short-term increases or decreases in sedentary behaviour are unlikely to harm or benefit this population’s risk of falls or fall-related injuries. It could be helpful to revisit this question with the CLSA data in the future to assess the impact of sedentary behaviour change over a longer duration.

This study has some limitations that must be noted. Firstly, in the PASE questionnaire, daily sedentary behaviour was capped at four hours. This creates an unbalanced weighting of participants in the ‘High’ sedentary condition and may have adversely affected the reporting of sedentary behaviour. The categories created to answer the research question are arbitrary and were directed by the tool and the exposure distribution. Future research would benefit from a more precise method of reporting sedentary behaviour (i.e., wearable device), particularly in aging adults, where it is more common (30). Secondly, the number of medical conditions and medications was reported and controlled. However, some medications will more substantially affect fall risk and fall-related injury than others. Without specific details of these medications, we cannot accurately determine their impact on the relationship between falls and sedentary behaviour. In addition, Fall-related injuries were collected through different questionnaires at baseline and follow-up. This may explain why fewer people recorded falling at follow-up.

Based on the results of this study, health practitioners should recommend limiting sedentary behaviour as young as possible to reduce the relative risk of falls and fall-related injuries. Future research in this area should focus on determining how much sedentary behaviour needs to be reduced and over what length to decrease the relative risk of injurious falls in middle—and older-aged adults.

## Conclusion

In conclusion, higher levels of sedentary behaviour are associated with a higher risk of fall-related injury independent of confounders in people aged 45 to 85. Considering this, older middle and older adults or those at risk of falling may benefit from less sedentary time. However, we found that changes in sedentary behaviour were not associated with injurious falls. Despite the results, a more precise measure of sedentary behaviour is needed for epidemiology studies to capture changes over time better.
